# Classification of *Lupinus* seeds into sweet and bitter categories using VIS–NIR spectroscopy and machine learning

**DOI:** 10.3389/frai.2026.1745720

**Published:** 2026-02-27

**Authors:** Josefa Díaz-Álvarez, Francisco A. Galea-Gragera, Francisco Chávez de la O, Pedro A. Salguero-López, Fernando Llera Cid

**Affiliations:** 1 Departamento de Tecnología de los Computadores y Comunicaciones, Centro Universitario de Mérida, Universidad de Extremadura, Mérida, Spain; 2 Pasture and Forage Crops Area, Finca La Orden-Valdesequera" Agricultural Research Institute, Extremadura Scientific and Technological Research Centre (CICYTEX), Badajoz, Spain; 3 Departamento de Ingeniería de Sistemas Informáticos y Telemáticos, Centro Universitario de Mérida, Universidad de Extremadura, Mérida, Spain

**Keywords:** absorbance spectra, artificial intelligence, food sustainability, resampling methods, seed phenotyping, spectral reflectance

## Abstract

**Purpose:**

The *Lupinus* germplasm includes sweet and bitter materials distinguished by compounds responsible for bitterness. Conventional identification is often destructive. This study assesses a non-destructive approach based on visible–near infrared (VIS-NIR) spectroscopy and machine learning to classify whole seeds from seven *Lupinus* species into sweet or bitter classes.

**Methods:**

Five machine-learning algorithms were evaluated on two datasets (reflectance and absorbance) acquired with VIS-NIR spectroscopy. Analyses were conducted on raw spectra and on spectra transformed using four spectral-transformation techniques. Because classes were imbalanced, five resampling methods were compared to improve classification performance.

**Results:**

Performance was assessed using *F1-score* and *ROC-AUC*. On reflectance, LGR and SVC reached 92.5 and 92.0%; on absorbance, SVC and RF achieved 93.2 and 92.5%. Hybrid transformations consistently improved discrimination, and resampling reduced overfitting associated with class imbalance.

**Conclusion:**

The results indicate that combining VIS–NIR spectroscopy with machine learning provides a suitable non-destructive alternative to discriminate sweet and bitter *Lupinus* materials/ecotypes.

## 1 Introduction

**Figure 1:**
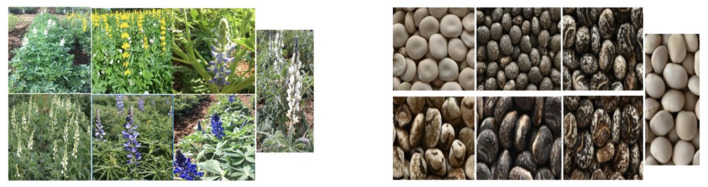
*Lupinus* species. Image credit: Francisco A. Galea-Gragera and Fernando Llera Cid.

Lupin is a legume of the genus *Lupinus* (Fabaceae), comprising more than 200 species. Its cultivation has been documented since antiquity, and species are native to the Mediterranean basin, North Africa, and the Americas (North and South). From these centers of origin, distribution later expanded to Asia, Australia, and Europe (Figure 1).

The process of domesticating lupins first took place in ancient civilisations. The factors that primarily drove it were its utility as a nutritional source, its capacity to enhance poor soils by fixing nitrogen, and its significant role in rotational growing cycles, which are crucial for a sustainable agricultural system. Since the second half of the 20th century, there has been an increasing interest in cultivating lupins, particularly for their nutritional potential, lack of genetic modification, sustainable intake, health benefits, and lower-cost (Boukid and Pasqualone, 2022; Johnson et al., 2017; Valente et al., 2024).

*Lupinus* seeds have a high protein content (36%–42%), comparable to or exceeding soybean (36%–40%). They are used in bakery products, pasta, snack formulations, and cosmetics (Food Innovation, 2020). The combination of plant proteins, high fiber content, and low saturated fat has been associated with improved glycaemic control and reduced cardiovascular risk (Boukid and Pasqualone, 2022). Seeds also contain bioactive compounds—polyphenols, carotenoids, phytosterols, tocopherols, alkaloids, and peptides—with antioxidant, anti-inflammatory, antimicrobial, and anticancer activities, of potential public-health relevance (Mazumder et al., 2024). This portfolio of properties has likewise attracted interest from the pharmaceutical sector (Romeo et al., 2018).

Lupin alkaloids, particularly quinolizidine alkaloids, represent a natural defense mechanism; nevertheless, they are toxic to humans and their ingestion poses a significant health risk. Lupin alkaloid poisoning in humans can affect the nervous, circulatory, and digestive system (Salsano et al., 2025).

Alkaloid content underpins the operational classification of material as bitter (≈1%–2%) or sweet (<0.05%), which in turn guides feed vs. food uses. “Sweet” material is not naturally occurring but results from selection and breeding to reduce alkaloid concentration. Cultivated species with sweet cultivars include *Lupinus albus* (white lupin), *L. angustifolius* (blue or narrow-leaf), *L. luteus* (yellow), and *L. mutabilis* (tarwi). Even within these groups, alkaloid levels vary among varieties, and debittering—brine washing, cooking, fermentation, or ultrasound—is often required (EFSA Panel on Contaminants in the Food Chain (CONTAM) et al., 2019; Estivi et al., 2023).

Traditionally, sweet/bitter determination has relied on the quantification of alkaloids using destructive reference methods, including LC–MS/MS (Khedr et al., 2023), GC–MS (Romeo et al., 2018), Soxhlet/Randall extraction (Kniepkamp, 2024), and HPLC (Eugelio et al., 2023). In parallel, non-destructive techniques such as near-infrared spectroscopy (NIRS), FTIR spectroscopy, and hyperspectral imaging (HSI) have gained prominence. NIRS is a rapid and effective tool for agri-food quality control, including the indirect assessment of bitterness-related traits in *Lupinus* (Schwertfirm et al., 2024). FTIR provides high spectral resolution for functional-group identification (Mirza et al., 2023). Finally, HSI integrates spatial and spectral information for quality evaluation (Elmasry et al., 2012; Siche et al., 2016)

This research aims to classify *Lupinus* material as sweet or bitter using seed-level VIS–NIR spectra (reflectance and absorbance) and a range of machine-learning algorithms. The combination of VIS–NIR spectroscopy and machine learning provides a rapid, non-destructive approach to discriminating between sweet and bitter classes. We adopt a data-driven machine-learning/AI strategy with a proven track record across agri-food applications.

The use of artificial intelligence to address real-world problems has proven effective across multiple domains. Within AI, machine learning (ML) is particularly prominent due to its broad applicability and methodological reach. Precision agriculture still presents substantial methodological and operational challenges. In this setting, ML techniques provide analytical power and practical solutions to well-characterized problems.

Research on *Lupinus* follows this global trend, as reflected by an increasing number of publications. A prominent line of work addresses the detection of invasive lupins (Sabat-Tomala et al., 2021, 2024; Schulze-Brüninghoff et al., 2021). For instance, Danilevicz et al. (2023) developed a deep-learning model operating on segmented images to identify sandplain lupins, particularly narrow-leaf lupins, achieving 80.3% performance. In Wijesingha et al. (2020), a Random Forest classifier discriminated lupin vs. non-lupin images with 89% mean accuracy. A comprehensive systematic review is provided by Singh et al. (2024). Furthermore, Petropoulos et al. (2025) evaluated six ML models for crop-yield prediction using Sentinel-2 data, with XGBoost performing best (*R*^2^ = 87.56%).

Evidence on lupin classification remains limited. In Coïsson et al. (2011), *L. albus* and *L. angustifolius* were discriminated by applying principal component analysis (PCA) and self-organizing maps (SOM) to chemical parameters; the first three components explained 76.3% of the variance. In Freire Diaz et al. (2021), an artificial-vision system was proposed to classify “sweet” *L. mutabilis* grains based on color and shape features.

Sweet–bitter discrimination has predominantly relied on alkaloid quantification using destructive analytical methods. Most studies employ GC–MS, LC–MS/MS, or related techniques. For example, Kroc et al. (2017) assessed quantitative and qualitative alkaloid composition in 367 *L. albus* ecotypes from the Polish Gene Bank, revealing marked differentiation associated with domestication. In Lee et al. (2020), quinolizidine alkaloids were quantified in three lupin products (beans, cookies, and a beverage) to evaluate consumer safety. Furthermore, Khedr et al. (2023) applied LC–MS/MS to five *Lupinus* species from a single farm targeting six alkaloids, and Khedr et al. (2024) compared the alkaloid content of commercial narrow-leafed lupin with wild ecotypes.

In Madelou et al. (2024) developed a methodology for quantifying simultanously eight major lupin alkaloids using quantitative NMR spectroscopy (qNMR) as an efficient and rapid analytical tool to detect variations in alkaloids content between species and subespecies. Recently, Sharma et al. (2024) presented a complete literature review about the most latest advancements and methodologies for detection and separation of alkaloids. Saini et al. (2024) also published the latest update of analytical methods for bioactive alkaloids.

Recently, Eugelio et al. (2024) proposed an approach based on UHPLC-QqQ-LIT-MS/MS data to detect the alkaloid profile. They presented an unsupervised learning proposal using Hierarchical Cluster Analysis (HCA) and a supervised approach with partial least squares discriminant analysis (PLS-DA) to classify samples according to their geographical origin. In a similar way, Namdar et al. (2024) used data provided by LS-MS/MS to identify the alkaloid profile using an unsupervised approach with HCA to relate lupin species and their QA. After a comprehensive literature review in Scopus and Google Scholar, it is worth to mention these two previous studies are just a few of the research works found that includes a supervised and/or unsupervised learning perspective to address some kind of classification of *Lupinus*. This highlights the originality of the research work, which aims to discriminate between bitter or sweet *Lupinus* based on spectral data collected from seven different *Lupinus* species.

## 2 Materials and methods

**Figure 2:**
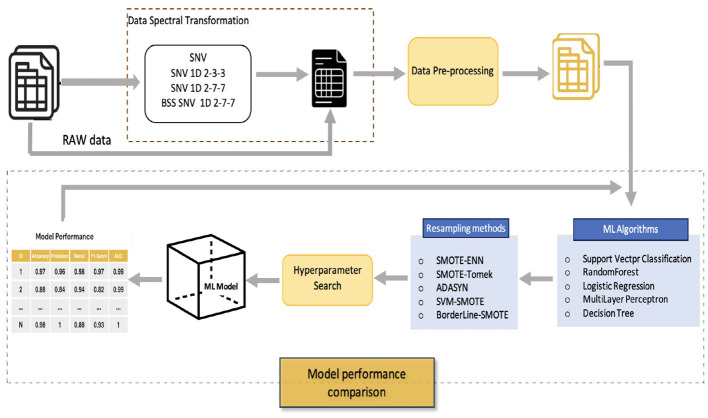
General scheme depicting the methodological phases to reach our research objectives.

This section describes the methodology and data used to achieve the research objectives. The methodological workflow is shown in Figure 2. The subsections are organized as follows: (i) plant material and data acquisition; (ii) preprocessing, including a brief summary of spectral transformations and resampling methods used to address class imbalance; and (iii) machine-learning techniques evaluated in this study.

### 2.1 Materials

This research work assessed seven annual species of lupin, including the three most widely cultivated species—*Lupinus albus* L. (white lupin), *Lupinus angustifolius* L. (blue lupin), *Lupinus luteus* L. (yellow lupin)—and four wild species: *Lupinus hispanicus* Bois and Reut, *Lupinus gredensis* Gand, *Lupinus micranthus* Geus, and *Lupinus cosentinii* Guss. The seeds were selected from the official active collection of the genus *Lupinus* at the CICYTEX Germplasm Bank, located at the “Finca La Orden-Valdesequera” Agricultural Research Institute (+38°51′2.5^′′^, −6°40′14.7^′′^; Figure 1). Conservation of ecotypes is conducted in a temperature-controlled environment, where the temperature is carefully regulated between 0 and 2° and 30% relative humidity to ensure the optimal conditions (Galea-Gragera and Llera Cid, 2025). Before analysis, seeds were acclimatized to room temperature to homogenize their moisture content and ensure representativeness in the spectral measurements.

#### 2.1.1 Assignment of sweet and bitter classes

The classification of accessions as sweet (low alkaloid content) or bitter (high alkaloid content) was obtained from the characterization and evaluation dataset associated with the Official Active Collection of grain *Lupinus* maintained at the CICYTEX Germplasm Bank. This information is managed independently from passport data (MCPD) and is assigned at the accession level (accename), following standard procedures commonly applied in germplasm banks and plant breeding programmes. In this context, material classified as sweet generally refers to seeds with a total quinolizidine alkaloid (QA) content below commonly accepted safety thresholds (approximately 0.02%–0.05% on a dry weight basis). These thresholds were established in previous studies using quantitative analytical techniques such as gas chromatography–mass spectrometry (GC–MS) and liquid chromatography–mass spectrometry (LC–MS) (Barzaghi et al., 2025; Engel et al., 2022; Namdar et al., 2024).

The sweet/bitter distinction constitutes a classical functional descriptor in the genus *Lupinus*, as quinolizidine alkaloids are the main secondary metabolites responsible for the characteristic bitterness and potential toxicity of the seeds. This criterion has been historically used in germplasm conservation, characterization, and breeding activities (Frick et al., 2017). In the specific case of the CICYTEX collection, this classification has been established through rapid qualitative alkaloid screening based on the Dragendorff test. This test is a classical colorimetric method widely used for the preliminary discrimination between low- and high-alkaloid *Lupinus* materials in germplasm bank and selection contexts (Barzaghi et al., 2025; Frick et al., 2017)

In the present study, the sweet/bitter labels were used as reference classes derived from official characterization records, without performing additional destructive chemical analyses. This decision was made because the primary objective of the work was to evaluate the potential of VIS–NIR spectroscopy combined with machine learning techniques as a rapid and non-destructive classification approach for screening and discriminating sweet and bitter material, rather than to replace the quantitative chemical determination of quinolizidine alkaloids by reference analytical techniques such as GC–MS or LC–MS (Barzaghi et al., 2025; Namdar et al., 2024)

### 2.2 Methods

#### 2.2.1 Spectral data acquisition

Non-destructive analysis of seeds is performed primarily using near-infrared spectroscopy (NIRS); visible–near infrared (VIS–NIR) spectroscopy is also used and, when spatial as well as spectral information is required, hyperspectral imaging (HSI). In this study, a FieldSpec 3 spectroradiometer (ASD Inc.) was used to acquire VIS–NIR spectra in interactance–reflectance mode, covering 350–2, 500 nm and yielding 2,151 spectral variables. These spectra capture information related to color as well as physiological and biochemical traits. The spectral resolution was 3 nm (maximum half-width) at 700 and 10 nm (maximum full half-width) at 1,400 and 2,100 nm.

Each measurement series was preceded by the recording of the reference spectrum or white reference using a ceramic plate, a procedure repeated for every five samples. The recording of the reference spectra and *Lupinus* seeds was carry out using RS3 Spectral Acquisition Software (ASD Inc.) and the ASD Turntable^1^ (150 mm, 22 rpm) equipped with a halogen light source.

**Figure 3:**
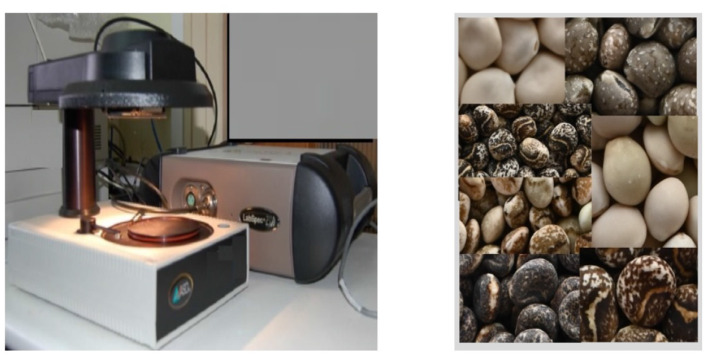
Equipment used for the capture and recording of spectral data: FieldSpec 3 spectroradiometer and ASD Turntable probe. Images on the right represent seed images of the seven *Lupinus* species included. Image credit: Francisco A. Galea-Gragera and Fernando Llera Cid.

Spectral measurements were acquired under controlled temperature, relative humidity, and lighting conditions. To prevent ambient-light interference, acquisitions were performed in darkness. The spectroscopy room of the Radiometry and Remote Sensing Laboratory (Forage Crops and Pastures Area) at the “Finca La Orden–Valdesequera” institute met all requirements. Figure 3 shows the FieldSpec 3 and ASD Turntable used.

It should be noted that the controlled temperature and relative humidity conditions refer to seed conservation prior to analysis in the germplasm bank facilities. Spectral acquisition was performed in a dedicated spectroscopy room under controlled lighting conditions (complete darkness) and stable laboratory temperature, following a standardized protocol developed at the Radiometry and Remote Sensing Laboratory of CICYTEX.

Each sample was analyzed in quadruplicate, and the spectrum was computed as the average of twenty-five consecutive scans. This procedure minimizes particle-size effects, improves the representativeness of the measurements, and increases data reproducibility and reliability (Gragera, 2015; Nicolaï et al., 2007).

Reflectance and absorbance define how materials interact with incident electromagnetic radiation. In this research work, both measurements were collected. The reflectance data (*R*) were directly collected from the FieldSpec 3 instrument. Absorbance spectra was obtained applying the standard formula *A* = −log_10_(*R*) for all spectral measurements. As a result, the reflectance and absorbance spectra are organized into two independent dataset.

#### 2.2.2 Preprocessing stage: data transformations

Raw spectra are affected by instrumental noise, environmental conditions, and sample complexity (light scattering, particle size), which degrade data quality and may mask relevant patterns. Consequently, spectral preprocessing is critical to ensure reliable analysis and improve the robustness of ML models (Kotsiantis et al., 2006).

Preprocessing operated on the averaged spectra obtained during acquisition (Section 2.2.1), which reduces instrumental and sample-related noise and supports robust downstream analysis (Nicolaï et al., 2007). As a first step, a quality-control check for missing values was performed, where none were found. The final dataset comprised 871 spectra, acquired in accordance with the procedure described in Section 2.2.1. Next, transformations were applied to mitigate the impact of signal-to-noise ratio issues, distortions, scattering, and baseline/background effects in the spectra (Miller, 2010).

The selection of techniques and their combinations was guided by the literature and by empirical tuning on the data (Grisanti et al., 2018), as these pretreatments have been shown to improve accuracy, reveal spectral patterns, and facilitate interpretation (Mishra et al., 2020).

The transformation techniques considered were: standard normal variate (SNV, per-spectrum centering and scaling to correct scattering and slope), Savitzky–Golay first derivative (1D, baseline reduction and detail enhancement). This technique requires careful parameter tuning to avoid noise amplification, particularly when combined with SNV, and baseline-shift correction (BSS) with polynomial fitting (removal of systematic background trends).

The selection of the combinations of transformation techniques proposed in this research work, a brief description and justification (Bian, 2022; Rinnan et al., 2009) are shown below:

- SNV: it corrects for scattering effects. It is a simple and straightforward processing flow that is suitable for in-line applications or embedded devices where computational resources are limited.
- SNV+1D (2-3-3): SNV followed by first derivative (window 2-3-3) It is strongly recommended for spectra with narrow peaks, so the shortest window preserves spectral details without compromising the global metric.
- SNV+1D (2-7-7): SNV followed by first derivative (window 2-7-7). It represents a highly accurate alternative with one less stage, making it ideal for reducing processing time without compromising on robustness.
- BSS+SNV+1D (2-7-7): sequential correction: BSS, SNV, and first derivative (window 2-7-7). It is perfect for studies where correcting for drift is important and computer resources are not an issue due its accuracy and consistency.

Pretreatments were applied to both reflectance and absorbance, producing for each transformation technique a specific dataset; together with the raw (unpretreated) dataset, these constitute a series of independent datasets used in subsequent analyses. This structure facilitated systematic comparisons and assessment of each pretreatment's impact on the performance of ML algorithms for classifying *Lupinus* seeds as sweet or bitter.

Stratified *K*-fold cross-validation (*K* = 5) was applied to evaluate the classification performance on the reflectance and absorbance datasets. This partitioning reduces the risk of overfitting, particularly under class imbalance (López et al., 2022).

#### 2.2.3 Preprocessing: class balancing

**Figure 4:**
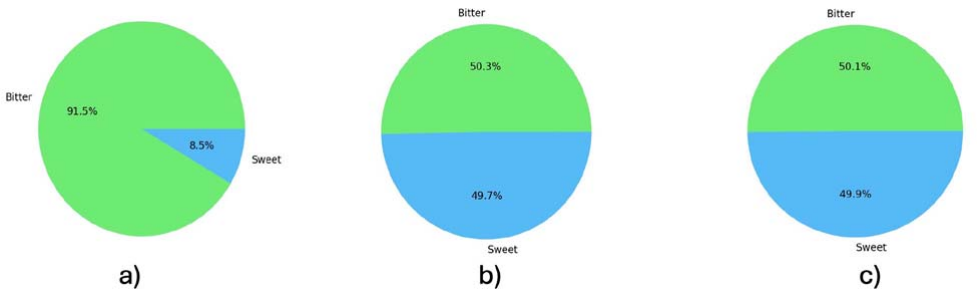
Frequency distribution of bitter/sweet *Lupinus* across both datasets (reflectance and absorbance). **(a)** Original data, *IR* = 10.77; after resampling: **(b)** SMOTE-ENN, *IR* = 1.02; **(c)** ADASYN, *IR* = 1.01. Distributions for the other methods are similar.

Class imbalance is a salient challenge in ML, particularly in real-world settings where classifiers tend to bias toward the majority class, increasing errors on the minority class and the overall misclassification cost (Kaur et al., 2019). Here, we address sweet vs. bitter discrimination using spectral information from ecotypes drawn from seven *Lupinus* species. The analysis revealed a non-uniform class distribution (Figure 4a), with under-representation of sweet material in both the reflectance and absorbance datasets.

The imbalance ratio (IR) is a simple and popular class imbalance measure that computes as the ratio of the number of samples in the majority class to the minority class (*IR* = *N*_*Instances*_*Majority*_/*N*_*Instances*_*Minority*_). The larger the IR, the more severe the imbalance issue (López et al., 2013). This measure was computed for the two datasets employed in this study, yielding a figure of *IR* = 10.77. The accuracy of ML classification is highly affected by imbalanced dataset. To rebalance the class distributions, reduce the imbalance ratio and improve the robustness of the models various resampling methods were evaluated. Resampling methods are statistical techniques that create new samples from the training set and gather more information about a sample, thereby improving the model accuracy (Elreedy et al., 2024; Wang et al., 2021). Figure 4 also shows the frequency distribution after applying two of the resampling methods as illustrative examples. Figure 4b for SMOTE-ENN and Figure 4c for ADASYN. The application of rest of the resampling methods resulted in a completed balanced datasets.

Table 1 summarizes the resampling methods applied and the IR resulting after their application. SMOTE-based methods and advanced techniques like ADASYN, that focuses on the hard-to-learn instances, were evaluated. For example, BorderLine SMOTE, SMOTE-Tomek, and SVM-SMOTE equalized the number of samples per class (IR = 1.00), while other methods, such as SMOTE-ENN and ADASYN are close to 1, SMOTE-ENN *IR* = 1.02, (c) ADASYN *IR* = 1.01.

**Table 1:** Description of the resampling methods applied in this research study and imbalanced ratio after resampling.

**Resampling tech**.	**Description**	**IR**
SMOTE-ENN	SMOTE Edited Nearest Neighbors. The minority class is oversampled using SMOTE, and then ENN is used to remove noisy or misclassified samples. This results in a dataset that is both more balanced and less noisy (Batista et al., 2004).	1.02
SMOTE-Tomek	SMOTE Tomek Links. Oversampling the minority class using SMOTE and undersampling with Tomek Links. TL identify pairs of samples from opposing classes that are each other's nearest neighbors and lie near the decision boundary. Eliminating the majority class instance from each Tomek Link enhances class separability and mitigates noise in imbalanced datasets (Batista et al., 2004).	1.00
SVM-SMOTE	SMOTE oversamples minority samples along the borderline and, then applies an SVM classifier for predicting new instances (Nguyen et al., 2011).	1.00
BL-SMOTE	BorderLine SMOTE. BL-SMOTE oversamples minority classes on borderline samples, instances that are often misclassified by their nearest neighbors (Han et al., 2005).	1.00
ADASYN	Oversampling focuses on instances that are harder to learn by adaptively assigning weights based on local data distribution (He et al., 2008).	1.01

#### 2.2.4 Machine learning techniques

Machine learning methods are widely used for their ability to identify complex patterns in data and produce accurate predictions. In this study, we evaluated five supervised algorithms to classify *Lupinus* materials/ecotypes as sweet or bitter from VIS–NIR spectral data. The classifiers compared were logistic regression (LGR), multilayer perceptron (MLP), support vector machine (SVC), random forest (RF), and decision tree (DTR). A brief description of each classifier is provided in Table 1.

Hyperparameters govern the learning process and strongly influence classifier performance. Selecting an appropriate combination is therefore critical to maximize accuracy. In this study, hyperparameter tuning was performed using GridSearchCV (grid search with cross-validation) from scikit-learn in Python. The large number of possible combinations results in considerable variability in outcomes, depending on the algorithm, transformation technique, and resampling method. GridSearchCV trains and evaluates the model via *K*-fold cross-validation, averaging performance across folds. This ensures robust, generalizable hyperparameter selection and allows control over model complexity. The set of hyperparameters and ranges evaluated for each algorithm and each preprocessing combination is provided in Table 2. The optimal values selected for each ML algorithm, data-transformation technique, and resampling method were fixed for the subsequent experiments. To reduce the risk of overfitting under a high-dimensional setting (2,151 wavelengths) relative to sample size, the hyperparameter search space was explicitly defined to control model complexity. For Logistic Regression, the inverse regularization strength (C) and the regularization scheme (e.g., L2 penalty) directly constrain coefficient magnitude, limiting overly flexible decision boundaries. For Random Forests and Decision Trees, complexity was controlled through parameters such as maximum tree depth (max_depth), minimum samples per split (min_samples_split), and minimum samples per leaf (min_samples_leaf), which restrict tree growth and reduce variance. For SVC with RBF kernels, C, and gamma jointly regulate the margin and the smoothness of the decision function. For MLP, architectural choices (hidden layer sizes) and regularization (alpha) constrain capacity. The ranges explored for these hyperparameters are reported in Table 3, and the selected values reflect the best cross-validated performance within the bounded search space.

**Table 2:** Description of the ML techniques evaluated to classify *Lupinus* seeds as sweet/bitter.

**ML tech**.	**Description**
LGR	It estimates the probability that a sample belongs to a specific class by applying a logistic regression function. It is suitable for binary and multi-class classification.
MLP	It is a particular type of artificial neural network designed for classification tasks. It consists of one input layer, one or more hidden layers and an output layer. Non-linear activation functions such as sigmoid or relu are used by Hidden Layers to model complex, non-linear relationships and to classify data.
RF	It is a widely used ML algorithm for classification and regression. It combines multiple decision trees based on the idea of ensemble learning, where each tree defines a model that is trained using samples that are randomly chosen and then predicts. The final prediction is decided by majority voting.
SVC	It is a variant of the support vector machine (SVM) that has been specially designed for classification tasks. It is based on support vectors, which are used to find the hyperplane that most effectively separates the classes in the data
DTR	It is a hierarchical tree structure made up of a root node, branches, internal decision and leaf nodes. DTR recursively splits the data based on the feature values using a defined criterion (Gini impurity or information gain) until the stopping condition is met. Internal nodes apply split rules based on feature values, which in turn generates new branches. The leaf node identifies the final decision.

LGR, logistic regression; MLP, multilayer perceptron; RF, random forest; SVC, support vector classification; DTR, decision tree.

**Table 3:** Hyperparameters and their value ranges explored in the hyperparameter tuning process for each algorithm.

**ML tech**	**Final hyperparameter values**
LGR	C: 0.001, 0.01, 0.1, 1, 10, 100, 1,000; penalty: l1, l2; solver: newton-cg, lbfgs, liblinear, sag, saga
MLP	hidden_layer_sizes: 10, 20, (10, 10); activation: logistic, relu; solver: sgd, adam; alpha: 0.0001, 0.05; learning_rate: adaptive; max_iter: 200
RF	n_estimators: 50, 100, 200, 500; max_depth: none, 10, 20, 30; min_samples_split: 2, 5, 10; min_samples_leaf: 1, 2, 4; max_features: sqrt, log2; bootstrap: true, false
SVC	Kernel: rbf, poly; C: 0.01, 0.1, 1, 10, 100, 1,000; gamma: auto, scale
DTR	Criterion: gini, entropy, log_loss; max_depth: none, 10, 20, 30, 50; min_samples_split: 2, 5, 10, 20; min_samples_leaf: 1, 2, 4, 10; max_features: sqrt, log2, none; splitter: best, random

## 3 Experimental results

This section presents the experimental framework and classification results for machine-learning models developed to discriminate sweet and bitter *Lupinus* materials/ecotypes using VIS–NIR reflectance and absorbance spectra from whole seeds. The performance of five ML algorithms was evaluated using spectral data as input.

Spectral data were analyzed either in raw form or after applying one of four spectral transformations; subsequently, five resampling methods were applied to handle class imbalance.

This experimental framework involves the comparison and identification of spectral transformations and resampling methods aimed at optimizing the analysis for each algorithm and the study objective. All feasible combinations were evaluated using F1-score and AUC–ROC, metrics appropriate for imbalanced datasets. F1-score harmonizes precision and recall (Equation 1); AUC–ROC is standard for binary classification and quantifies the model's discriminative ability.


$$\begin{array}{l} F1-Score=2\times \left(precision\times recall\right)/\left(precision+recall\right) \end{array}$$ (1)

The results obtained with the stratified *K*-fold cross-validation (*K* = 5) strategy proposed for reflectance and absorbance data are outlined in the next subsection. All information refer to the test results.

Before presenting the classification performance metrics, a dedicated spectral analysis was conducted to characterize the intrinsic differences between bitter and sweet *Lupinus* seeds at the signal level. This analysis is independent of any machine learning model and aims to (i) identify systematic spectral contrasts inherent to the seed material; and (ii) provide a physically grounded basis for the interpretation of subsequent classification results and model-derived spectral importance.

### 3.1 Spectral differentiation and selectivity analysis

#### 3.1.1 Exploratory spectral comparison between bitter and sweet seeds

Before evaluating supervised classification performance, this section explores which spectral regions contribute to the natural variability between seeds labeled as *bitter* and *sweet*. In this light, a spectral difference analysis was computed as $\Delta R\left(\lambda \right)={\bar{R}}_{\text{bitter}}\left(\lambda \right)-{\bar{R}}_{\text{sweet}}\left(\lambda \right)$ using raw reflectance spectra in the 350–2,500 nm range. This approach highlights systematic contrasts in the mean spectral signature between classes, which may be partially masked in individual spectra due to the strong spectral collinearity that characterizes VIS–NIR data (Roggo et al., 2007; Weyer and Lo, 2006).

**Figure 5:**
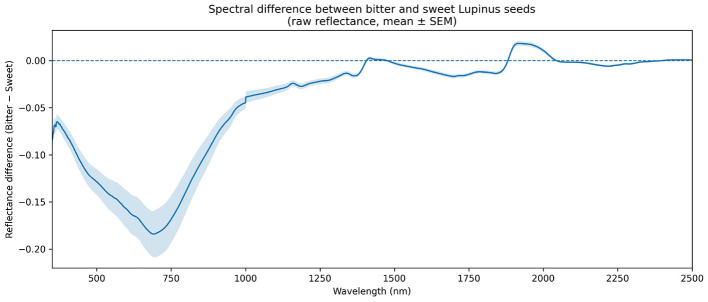
Spectral difference between *bitter* and *sweet Lupinus* seeds based on raw VIS–NIR reflectance data (350–2,500 nm). The solid line represents Δ*R*(λ) (Bitter−Sweet), and the shaded band indicates ± SEM at each wavelength. Negative values indicate higher reflectance in *sweet* seeds, whereas positive values correspond to higher reflectance in *bitter* seeds.

Figure 5 shows the spectral difference profile together with the ± standard error of the mean (SEM) band at each wavelength. This representation enables the magnitude of the class contrast and its relative stability against within-class variability to be assessed simultaneously (Sem, 2021). Consequently, it facilitates the identification of wavelength regions where the separation between *bitter* and *sweet* exceeds internal dispersion, suggesting structured spectral differences rather than instrumental noise.

Overall, the results reveal consistent spectral differences across the VIS–NIR domain. In the visible region (approximately 400–700 nm), *sweet* seeds tend to exhibit higher reflectance than *bitter* seeds, which is compatible with variation in seed coat color and the contribution of pigment- and phenolic-associated compounds (Weyer and Lo, 2006). In the near- and short-wave infrared range (700–2,500 nm), subtler but persistent differences are observed, linked to overtone and combination bands of molecular vibrations. In particular, contrasts in regions commonly associated with C–H, N–H, and O–H absorptions are consistent with differences in the overall chemical composition of the seed matrix (organic and nitrogen-containing constituents), thereby reflecting an *intrinsic physicochemical signature* of the material rather than effects driven by data processing or algorithmic decisions (Beć et al., 2025; Weyer and Lo, 2006).

Taken together, the spectral difference analysis confirms the presence of systematic and structured contrasts between *bitter* and *sweet* seeds at the VIS–NIR signal level, providing a physical basis for the application of supervised classification methods. However, this descriptive analysis does not directly indicate which spectral regions are effectively exploited by machine learning models during the discrimination process. To enhance interpretability of the observed performance and to link spectral information with classifier behavior, a complementary spectral selectivity analysis based on an interpretable linear model was therefore conducted.

#### 3.1.2 Spectral selectivity based on logistic regression coefficients

**Figure 6:**
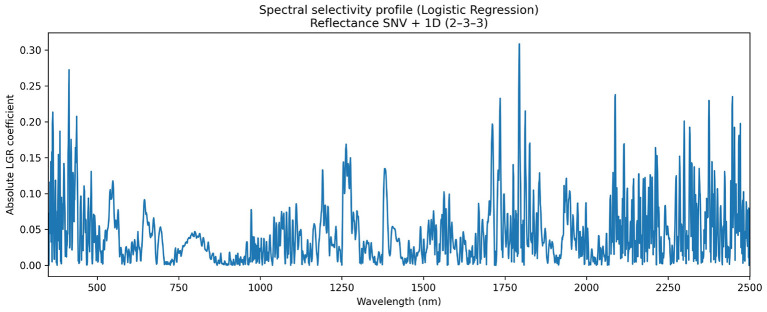
Spectral selectivity profile derived from a Logistic Regression (LGR) model trained on reflectance spectra preprocessed with SNV and first-derivative Savitzky–Golay filtering (2-3-3). The curve represents the absolute value of the model coefficients for each wavelength, indicating the relative contribution of spectral regions to bitter/sweet discrimination in *Lupinus* seeds. Highlighted peaks suggest regions of delocalised molecular information linked to global compositional differences in the seed matrix (Beć et al., 2025).

To identify the spectral regions underlying bitter/sweet discrimination, a spectral selectivity analysis was conducted using the coefficients of a Logistic Regression (LGR) model trained on reflectance spectra preprocessed with Standard Normal Variate (SNV) and first-derivative Savitzky–Golay filtering (2-3-3) Figure 6. This configuration was selected because it provided the highest classification performance for LGR under reflectance data. Spectral variables were previously standardized to ensure comparability across wavelengths, and selectivity was represented as the absolute value of the coefficient associated with each wavelength, assuming that in linear models each coefficient reflects the direct contribution of its corresponding feature to the prediction (Contreras and Bocklitz, 2025).

The resulting selectivity profile exhibits informative contributions distributed across the entire VIS–NIR range. Local maxima of importance were identified in the visible region (approximately 413–414 nm), associated with electronic transitions (Yang and Mouazen, 2012), and in three short-wave near-infrared regions (approximately 1,735–1,795, 2,087–2,088, and 2,375–2,447 nm), corresponding to overtone and combination vibrations of C–H functional groups (Weyer and Lo, 2006). This pattern indicates that discrimination does not rely on isolated spectral bands, but rather emerges from delocalised contributions along the wavelength axis, which is consistent with the multivariate nature of NIR signals and the complexity of non-fundamental vibrational transitions (Beć et al., 2025; Weyer and Lo, 2006).

### 3.2 Reflectance results

**Figure 7:**
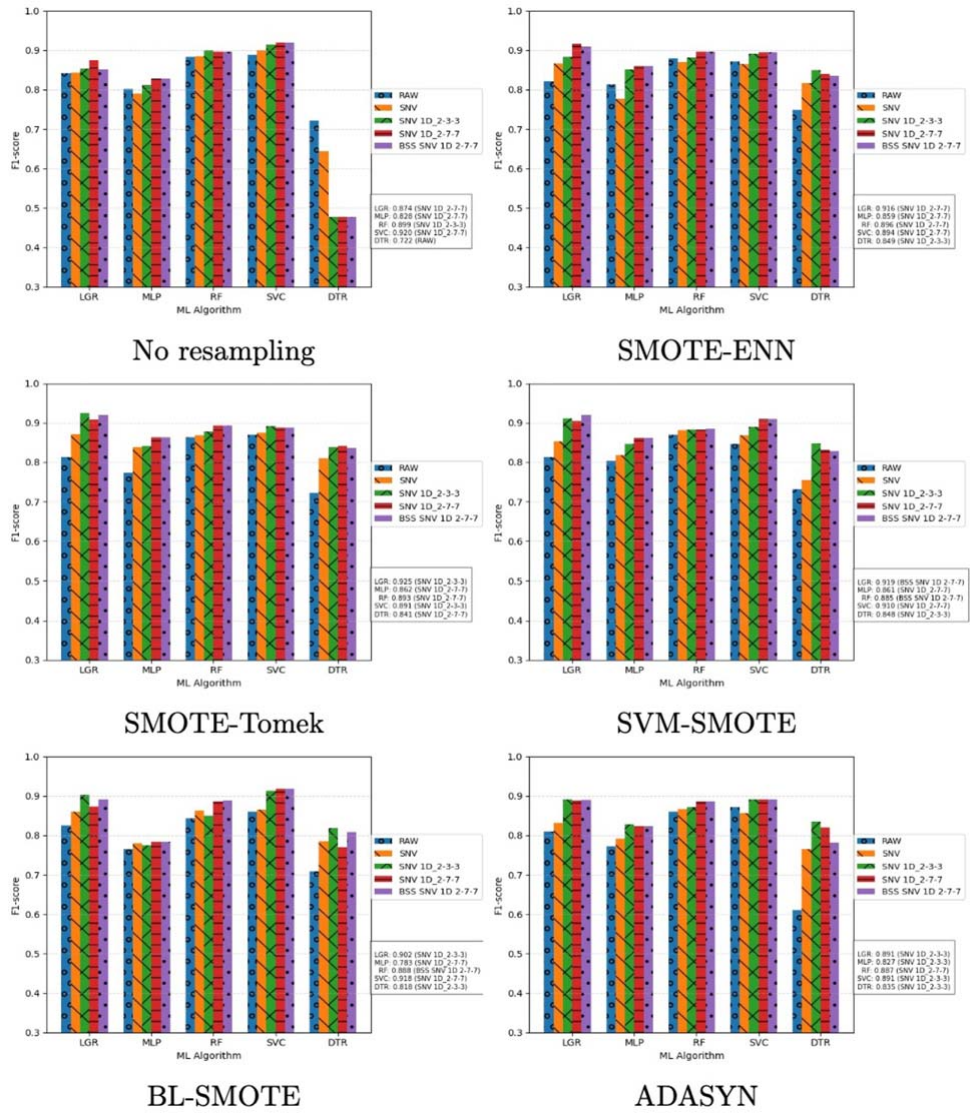
Performance of ML classifiers on VIS–NIR reflectance assessed by F1-score. Each plot corresponds to a resampling technique; the *x*-axis lists the classifiers and the *y*-axis shows F1-score averaged over five fold Each transformation technique is represented with a bar and identified according to a color code, which is shown on the right. For each classifier, the best F1-score and the associated transformation are reported on the right.

This subsection presents results for VIS–NIR reflectance. Performance was evaluated using F1-score and AUC–ROC. Figure 7 shows one plot per resampling method; bar colors encode the transformation techniques. The *x*-axis lists the classifiers, and the *y*-axis shows F1-score, computed as the mean over five fold. For each classifier, the maximum F1-score and the associated transformation are reported on the right.

Examining per-classifier maxima, the highest F1-scores were obtained predominantly with hybrid transformations, notably SNV+1D (2-3-3) and SNV+1D (2-7-7). RF and SVC performed consistently well across transformations: for RF, maxima ranged from 88.5% (SVM–SMOTE) to 89.9% (no resampling); for SVC, from 89.1% (ADASYN and SMOTE–Tomek) to 91.8% (BL–SMOTE). LGR achieved the overall best result (92.5%) with SMOTE–Tomek; across other resampling methods it ranged from 87.4% (no resampling) to 91.9% (SVM–SMOTE). Under reflectance, LGR emerges as the most effective option for sweet vs. bitter discrimination with four of five resampling methods.

In general, MLP and DTR classifiers showed the worst performance. However, while MLP exhibited acceptable scores ranged from 78.3% (BL-SMOTE) to 86.2% (SMOTE-Tomek), DTR reached the poorest scores ranged from 72.2% (No resampling) to 84.9% (SMOTE-ENN). It is noteworthy that both showed remarkable improvements when hybrid data transformation techniques were applied, as illustrated the results in Figure 7.

**Figure 8:**
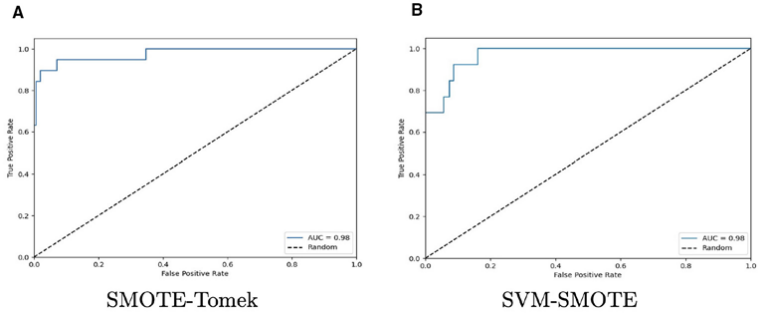
ROC curves for LGR with spectral reflectance data for SMOTE-Tomek **(A)** and SVM-SMOTE **(B)** as resampling methods and SNV+1D (2-3-3) and BSS+SNV+1D (2-7-7) as data transformation techniques, respectively. Both were chosen for exhibiting the best performance scores.

As previously mentioned, the AUC-ROC metric was also computed. As LGR was the classifier with the highest scores using spectral reflectance data and using SMOTE-Tomek and SVM-SMOTE as resampling methods, the AUC-ROC analysis is focused on this two methods as they demonstrated similar levels of performance for all classifiers and spectral transformation techniques. First, Table 4 outlines AUC values and standard deviation. Figure 8 plots ROC curves for LGR with both resampling methods and SNV+1D (2-3-3) and BSS+SNV+1D (2-7-7) as data transformation technique. They were chosen as an example of its behavior to discriminate between bitter and sweet material *Lupinus*. ROC curves plot the True Positive rates (TPR) against the False Positive rates (FPR), which provide with a graphical representation of the performance of a binary classifier at different classification thresholds. The summary of the performance is represented with the AUC value.

**Table 4:** AUC values (± standard deviation) of test for ML classifiers and data transformation techniques with SMOTE-Tomek and SVM-SMOTE as resampling methods on VIS–NIR reflectance.

**Transformation**					
**technique**	**LGR**	**MLP**	**RF**	**SVC**	**DTR**
**AUC values - SMOTE-Tomek**					
RAW	0.965 (±0.020)	0.798 (±0.136)	0.959 (±0.021)	0.966 (±0.017)	0.894 (±0.113)
SNV	0.950 (±0.047)	0.858 (±0.053)	0.968 (±0.018)	**0.981** (±0.011)	0.959 (±0.027)
SNV+1D (2-3-3)	0.978 (±0.018)	0.880 (±0.062)	0.975 (±0.018)	0.961 (±0.022)	0.965 (±0.020)
SNV+1D (2-7-7)	0.975 (±0.003)	**0.894** (±0.023)	**0.976** (±0.003)	0.970 (±0.003)	0.964 (±0.005)
BSS+SNV+1D (2-7-7)	**0.981** (±0.024)	**0.894** (±0.053)	**0.976** (±0.018)	0.970 (±0.028)	**0.966** (±0.020)
**AUC values - SVM-SMOTE**					
RAW	0.963 (±0.023)	0.852 (±0.097)	0.961 (±0.018)	0.961 (±0.020)	0.939 (±0.045)
SNV	0.941 (±0.045)	0.868 (±0.088)	0.971 (±0.020)	**0.978** (±0.014)	0.948 (±0.034)
SNV+1D (2-3-3)	0.972 (±0.026)	0.877 (±0.049)	0.975 (±0.018)	0.976 (±0.017)	**0.969** (±0.020)
SNV+1D (2-7-7)	**0.981** (±0.016)	**0.891** (±0.041)	**0.977** (±0.018)	0.974 (±0.024)	0.965 (±0.017)
BSS+SNV+1D (2-7-7)	0.938 (±0.011)	**0.891** (±0.041)	**0.977** (±0.018)	0.974 (±0.024)	0.968 (±0.017)

Both metrics provide similar performance results and behavior. The best values were highlighted in bold.

### 3.3 Absorbance results

**Figure 9:**
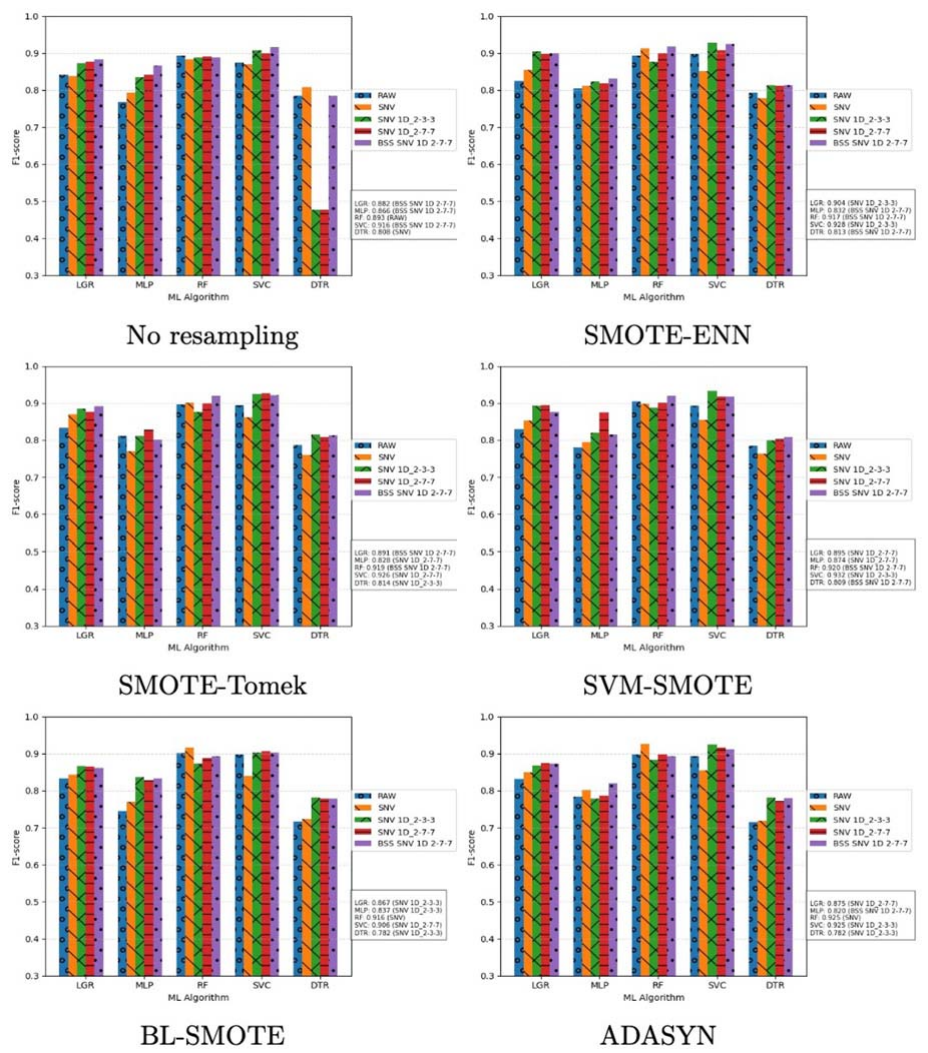
Performance assessment for each ML classifier with spectral absorbance data using the F1-Score metric. Every plot shows the results for a resampling methods. *X*-axis represents the ML classifiers and *Y*-axis is the F1-Score averaged over five fold. Each transformation technique is represented with a bar and identified according to a color code, which is shown on the right. The best score for each classifier and transformation strategy is depicted on the right.

This section analyses the experimental results obtained with VIS–NIR absorbance data. Figure 9 shows a summary focused on the F1-score. This analysis includes an independent graph for each resampling method, where the evolution of the performance of the different classifiers and data transformation technique is outlined. All graphs provide on the right a summary with the best F1-score value for each classifier and transformation strategy.

It is noteworthy that absorbance results confirmed the pattern observed with reflectance. In general, hybrid transformations yielded performance gains. The only exception was DTR, which deviated from this trend on the raw data.

Moreover, all experimental outcomes, when contrasted with the reflectance results, confirmed that RF and SVC exhibited the best performance and LGR presented a good performance, but it never was too close to RF and SVC. In this regard, RF exhibited its lowest performance with original data 89.3% and ranged from 91.6% (BL-SMOTE) to 92.5% (ADASYN), which represent excellent scores in the research objective. SVC performance ranged from 90.6% (BL-SMOTE) and 93.2% (SVM-SMOTE). Therefore, SVC was the classifier with the highest F1-score. Both algorithms confirmed that they are the most appropriate classifiers for VIS–NIR absorbance data, demonstrating consistent and relevant results with both original and transformed data, and throughout all resampling methods.

With respect to LGR, identified as the best classifier under reflectance, performance was solid across all experiments, with F1-score between 86.7% (BL–SMOTE) and 90.4% (SMOTE–ENN). Although these values were slightly lower than those of RF and SVC, LGR remains competitive on VIS–NIR absorbance data.

In accordance with reflectance, MLP and DTR classifiers showed the worst performance with absorbance. However, while MLP performed reasonably well with scores ranging from 82.0% (ADASYN) to 87.4% (SVM-SMOTE), which were higher than the scores obtained with spectral reflectance, DTR reached the poorest scores ranging from 78.2% (ADASYN and BL-SMOTE) to 81.4% (SMOTE-Tomek). Once more, both exhibited enhancements when hybrid data transformation techniques were implemented, as outlined in Figure 9. Nevertheless, only DTR with no resampling did not follow this trend, which is an interesting case to be further studied.

**Figure 10:**
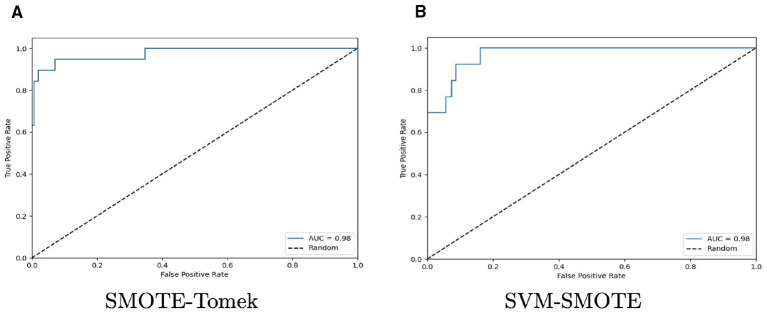
ROC curves for RF with ADASYN **(A)**, and SVC with SVM-SMOTE **(B)** and spectral absorbance data. SNV and SNV+1D (2-3-3) as data transformation techniques for RF and SVC, respectively. Both were chosen for exhibited the best performance scores.

These results were also analyzed using the ROC-AUC metric to depict the discrimination ability of a binary classifier between bitter and sweet *Lupinus* materials/ecotypes. After comparing the results, SVM-SMOTE and ADASYN were selected as the resampling methods with the highest performance for RF and SVC. Therefore, Table 5 summarizes the AUC values and the standard deviation for all classifiers and data transformation techniques for resampling methods chosen. Additionally, Figure 10 plots ROC curves for RF and SVC using ADASYN and SVM-SMOTE, respectively. Both were chosen because RF and SVC reached their highest performances with the application of these resampling methods.

**Table 5:** AUC values (± standard deviation) for ML classifiers and data transformation techniques with ADASYN and SVM-SMOTE as resampling methods using spectral absorbance data.

**Transformation**					
**technique**	**LGR**	**MLP**	**RF**	**SVC**	**DTR**
**AUC values - ADASYN**					
RAW	0.966 (±0.026)	0.820 (±0.094)	**0.977** (±0.044)	0.976 (±0.027)	0.955 (±0.031)
SNV	0.944 (±0.056)	0.882 (±0.075)	**0.977** (±0.045)	**0.982** (±0.020)	0.958 (±0.030)
SNV+1D (2-3-3)	0.967 (±0.045)	0.801 (±0.087)	0.966 (±0.041)	0.976 (±0.046)	0.962 (±0.027)
SNV+1D (2-7-7)	0.969 (±0.043)	**0.890** (±0.023)	**0.977** (±0.041)	0.980 (±0.040)	0.968 (±0.029)
BSS+SNV+1D (2-7-7)	**0.972** (±0.032)	0.862 (±0.051)	**0.977** (±0.047)	0.972 (±0.051)	**0.970** (±0.034)
**AUC values - BL-SMOTE**					
RAW	0.965 (±0.026)	0.820 (±0.089)	**0.978** (±0.044)	0.977 (±0.031)	0.957 (±0.031)
SNV	0.937 (±0.091)	0.817 (±0.055)	0.976 (±0.047)	**0.982** (±0.021)	0.958 (±0.035)
SNV+1D (2-3-3)	0.968 (±0.052)	0.848 (±0.062)	0.970 (±0.041)	0.978 (±0.042)	**0.972** (±0.031)
SNV+1D (2-7-7)	0.973 (±0.043)	**0.908** (±0.048)	0.976 (±0.045)	0.979 (±0.041)	0.971 (±0.031)
BSS+SNV+1D (2-7-7)	**0.981** (±0.029)	0.841 (±0.088)	**0.978** (±0.045)	0.979 (±0.042)	**0.972** (±0.029)

These were chosen for providing similar performance results and behavior. The best values were highlighted in bold.

The complementary spectral differentiation and selectivity analyses provide interpretative support for the classification results obtained. The presence of consistent spectral differences between bitter and sweet seeds across the VIS–NIR range indicates that discrimination is not driven solely by algorithmic decisions, but is grounded on intrinsic spectral signals of the plant material. Moreover, the selectivity analysis based on an interpretable model shows that the information exploited by the classifier is distributed across multiple spectral regions, consistent with the combined contribution of overtone and combination bands associated with O–H, N–H and C–H bonds. In near-infrared spectroscopy, these spectral features are commonly related to global compositional variations in complex biological matrices rather than to single compounds (Manley, 2014; Roggo et al., 2007; Weyer and Lo, 2006). In this context, spectral selectivity adds an interpretative layer to the predictive results and reinforces the physico-chemical coherence of the proposed approach, without implying a one-to-one assignment between individual spectral regions and specific chemical constituents.

### 3.4 Statistical tests

Once the results of the studies carried out in this work have been presented, a statistical analysis must be performed to evaluate the quality and robustness of the presented techniques.

Given the combined use of spectral transformations and resampling, normality and homoscedasticity were assessed per fold using Shapiro–Wilk and Levene tests, respectively. For Shapiro–Wilk, *H*_0_ assumes normality and *H*_1_ non-normality; for Levene, *H*_0_ assumes equal variances and *H*_1_ that at least one variance differs. Based on these diagnostics, the global test was selected: ANOVA (parametric) or Kruskal–Wallis (non-parametric) to test *H*_0_ (all populations equal) vs. *H*_1_ (at least one different).

This more in-depth study will enable us to determine whether, given the results obtained in the tests for the F1-Score and AUC metrics, we can affirm that there are significant differences between the ML methods. Finally, we can conclude by performing a robustness test, which will allow us to determine the most robust model that has been optimized in the work presented.

The robustness test will facilitate the identification and quantification of the optimal solution to the problem addressed.

Statistical tables with the detailed results on VIS–NIR spectral absorbance and reflectance data using F1-score are reported in the Supplementary material for clarity. Analyses with AUC yielded similar results. The statistical study was conducted for each of the leading algorithms. Overalll, it confirms that, although minor performance differences can be observed among classifiers depending on the spectral transformation and resampling strategy, these differences are generally small (data presented in Supplementary Tables S1, S4). In both reflectance and absorbance datasets, SVC, RF and LGR consistently form the most robust group of classifiers, showing stable performance across preprocessing configurations and resampling methods. This result supports the use of these algorithms as reliable alternatives for sweet/bitter discrimination in *Lupinus* seeds.

### 3.5 Wavelength selection during sweet/bitter discrimination for a specific study case

**Figure 11:**
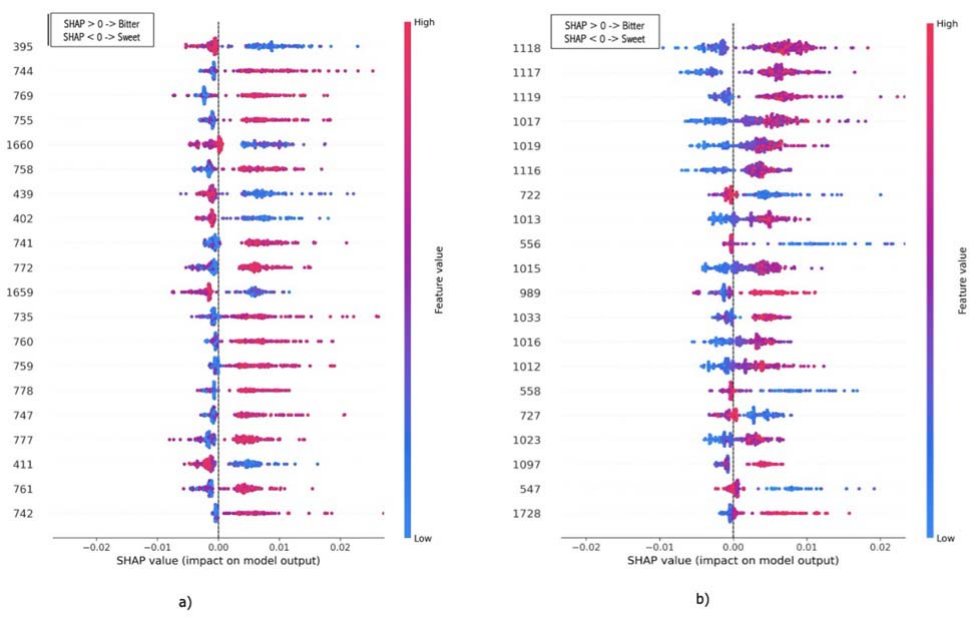
Feature importance during the test phase for reflectance **(a)** and absorbance **(b)** spectra. The most relevant 20 wavelengths were represented. The study case represents RandomForest as the ML algorithm, BSS-SNV-1D (1-7-7) and BL-SMOTE as the transformation technique and resampling method, respectively. The *Y*-axis indicates wavelength and the *X*-axis shows the SHAP value. Red color identifies bitterness and blue color means sweetness.

This section presents a succinct analysis of the most relevant spectral bands that were employed by RandomForest to discriminate between sweet and bitter *Lupinus*. BSS-SNV-1D (1-7-7) and BL-SMOTE were selected as the transformation technique and the resampling method, respectively, for reflectance and absorption spectra. Given the number of spectral variables (wavelengths), graphs in Figure 11 show the 20 most relevant wavelengths during the test phase. This provides a clear indication of which part of the spectrum was being used.

The SHapley Additive exPlanations (SHAP) value was used. SHAP explains predictions by measuring the contribution of each feature to deviations from the average model output, grounding in cooperative game theory.

UV (00–400 nm), VIS (400–700 nm), NIR (700–1,300 nm) and SWIR (1,300–2,500 nm) spectral regions capture information related to chemical composition and structure.

According to these results, the 20 most relevant wavelengths are distributed across the VIS, NIR, and SWIR regions. However, for reflectance data in this interval, there is a lower representation of the VIS and SWIR regions, and the relevant NIR wavelengths are concentrated near the lower limit of the NIR range. In contrast, for absorbance data, the most relevant NIR wavelengths are located closer to the upper limit of the NIR range. This selection used for RF to discriminate sweet/bitter seeds is in the line with the findings presented in Section 3.1.1.

## 4 Discussion

This study addressed the binary classification of *Lupinus* materials/ecotypes as sweet or bitter using VIS–NIR reflectance and absorbance spectra from whole seeds. The experimental design aimed to assess discriminative ability and to provide a non-destructive, supervised-learning approach. Five ML algorithms were compared on raw spectra and after four spectral transformations, together with five resampling methods to address class imbalance.

An initial exploratory spectral comparison stated that despite their relatively low abundance, recent work has shown that low-alkaloid material can be successfully discriminated using NIR spectroscopy even on intact seeds, leveraging matrix-level compositional differences captured by the spectral signature (Barzaghi et al., 2025). Moreover, the patterns of spectral differentiation, expressed as broad multivariate contrasts rather than isolated peaks, aligns with current recommendations for interpretation in applied spectroscopy and spectral machine learning, which emphasize linking predictive performance to physically plausible spectral evidence (Beć et al., 2025; Sem, 2021). Taken together, these findings support VIS–NIR spectroscopy as a non-destructive and interpretable tool for rapid germplasm screening (Barzaghi et al., 2025).

Regarding the selectivity analysis, it is important to note it was performed using original samples without synthetic resampling. Although class-balancing techniques such as SMOTE can improve statistical classification performance, in spectroscopy they generate artificial instances that do not represent real biological states of the seed. This may disrupt the physical covariance structure between delocalised variables, thereby distorting biochemical interpretation and model transparency (Beć et al., 2025; Contreras and Bocklitz, 2025).

In reference to the data transformation techniques, the combination of various transformations on the data available had a direct impact on the classifiers' performance, which have been identified as a general trend across the experimental results for all resampling methods and ML algorithms. With respect to the resampling methods evaluated, the results were quite consistent for all SMOTE-based techniques, ADASYN, even though with the raw data. Although some differences existed when the results of the classifiers were compared, variations in performance for the best classifiers were really narrowed.

Regarding the strategy used to identify optimal hyperparameter values and the risk of overfitting, the performance metrics on the training and test sets for the majority of the experiments showed differences generally between 1 and 5%, suggesting that most models generalize well and did not exhibit overfitting. Slightly larger differences were observed in test sets with few instances of the minority class, likely due to the higher variability inherent in small sample sizes. Nevertheless, further analysis may be required to more thoroughly assess model robustness and potential overfitting effects, particularly in imbalanced or low-sample settings.

In this regard, other alternatives for hyperparameter selection can be addressed using approaches such as randomized search or Bayesian optimization, which enable the identification of promising parameter combinations without exhaustively exploring the entire search space and reduce the risk of overfitting.

Focusing on classifier performance, three methods stood out with VIS–NIR reflectance and absorbance data: LGR, RF, and SVC. Under reflectance, LGR and SVC were prominent; under absorbance, RF and SVC were. In particular, LGR achieved the best result (92.5%) with SNV+1D (2-3-3) as the transformation and SMOTE–Tomek as the resampling method, on reflectance data. SVC ranked second, especially with raw data (92.0%) and with BL–SMOTE (91.8%) as the resampling method, in both cases using SNV+1D (2-7-7). RF exceeded 88.5% across all settings, constituting a robust alternative for the study objective.

Regarding VIS–NIR absorbance results, SVC exhibited the best performance across all experiments (90.6%–93.2%), consistently when hybrid transformation techniques were applied, demonstrating its potential to discriminate between sweet and bitter *Lupinus* material. RF ranked second, ranging from 89.3% on raw data without resampling to 92.5% with SNV and ADASYN as the transformation and resampling method, respectively. Although LGR did not achieve the top performance on absorbance, it achieved a good score (90.4%) with SNV+1D (2-3-3) and SMOTE–ENN.

It should be acknowledged that the high dimensionality of VIS–NIR spectra relative to sample size introduces an inherent risk of overfitting and may affect the stability of wavelength-level importance estimates. In this study, this risk was mitigated through stratified cross-validation, bounded hyperparameter search spaces with explicit complexity constraints, and the use of an interpretable linear model (logistic regression) for spectral selectivity. Moreover, spectral differentiation analyses were performed on raw data without model fitting, providing an independent, descriptive verification of structured class contrasts. Nevertheless, variable-importance patterns should be interpreted as indicative regions rather than unique causal wavelengths, and future work with larger and more balanced datasets, external validation, and/or formal wavelength selection will further strengthen the robustness of these interpretations.

## 5 Conclusions

According to the results with the data available in this study, we can confirm the potential of machine learning models as a non-destructive methodology to discriminate *Lupinus* sweet and bitter materials/ecotypes from VIS–NIR reflectance and absorbance spectra. LGR, RF, and SVC were identified as robust ML algorithms for both spectra data, with a classification performance higher than 90.4, 88.5, and 90.6%, respectively. Furthermore, hybrid classification techniques and well-known resampling methods have demonstrated their ability to improve this classification ability.

In addition, the spectral differentiation and selectivity analyses performed confirm that the classification between bitter and sweet *Lupinus* seeds relies on reproducible spectral patterns across the VIS–NIR range, further supporting the robustness and physical plausibility of the proposed non-destructive approach.

A partial analysis of the feature importance shows that although Random Forest feature importance does not provide a direct chemical interpretation, the results were consistent with those obtained from the spectral difference and selectivity analyses. This indicates that the classification between bitter and sweet *Lupinus* seeds relies on information distributed across VIS–NIR range, further supporting the robustnes of the proposed non-destructive approach.

Nevertheless, although the results obtained are significant and open up a non-destructive method, they must be interpreted cautiously until further analyses with a more balanced dataset are conducted.

Finally, future research should focus on two main aspects. The first one will be to achieve a more balanced dataset by increasing the number and diversity of samples in the minority class, in order to further improve model robustness and generalization. The second research direction will be to move beyond the preliminary identification of informative spectral regions toward formal wavelength selection and/or dimensionality reduction strategies, with the aim of simplifying the models and facilitating their implementation in portable devices or online/at-line classification systems. In this context, the identification of spectrally relevant wavelength regions may also provide guidance for future developments in problem-specific spectral sensing, although such applied perspectives remain outside the scope of the present study.

## Data Availability

The raw data supporting the conclusions of this article will be made available by the authors, without undue reservation.

## Footnotes

1
https://www.malvernpanalytical.com/
