# Supplementary material for: Classification of Lupinus seeds into sweet and bitter categories using VIS–NIR spectroscopy and machine learning
Source: Front Artif Intell. 2026 Feb 27;9:1745720. doi: 10.3389/frai.2026.1745720 (PMC12982427; doi:10.3389/frai.2026.1745720)
Supplement: Supplementary file 1 [file Data_Sheet_1.pdf]

Supplementary Material

1 SUPPLEMENTARY TABLES FOR STATISTICAL ANALYSIS

Tables S1 and S2 depicts the statistical tests applied to spectral absorbance data. The initial statistical evaluation, as outlined in Table S1, facilitates the identification of the most appropriate test to ascertain the substantial disparities between the optimized ML models. Finally, the table shows the significant differences between the ML models for each experiment. In cases where significant differences are detected (SD=*Yes*), we can say that the ML model with the best results can be considered the best solution.

Table S1. Statistical study based on F1-Score for Absorbance. Abreviations: Res. = Resampling technique, H = Kruskal-Wallis, and SD = Significant Differences.

Res.	Transform	Shapiro p-values	Levene p-value	Applied Test	p-value	SD
1	BSS SNV 1D 2-7-7	[0.451, 0.226, 0.379, 0.286, 0.045]	0.976	H	0.102	No
	RAW	[0.199, 0.268, 0.003, 0.844, 0.120]	0.985	H	0.012	Yes
	SNV	[0.228, 0.987, 0.410, 0.430, 0.559]	0.642	ANOVA	0.131	No
	SNV 1D 2-3-3	[0.370, 0.599, 0.407, 0.048, 0.314]	0.380	H	0.008	Yes
	SNV 1D 2-7-7	[0.007, 0.236, 0.070, 0.072, 0.31]	0.585	H	0.009	Yes
2	BSS SNV 1D 2-7-7	[0.777, 0.920, 0.163, 1.000, 0.343]	0.683	ANOVA	0.011	Yes
	RAW	[0.742, 0.515, 0.091, 0.097, 0.672]	0.913	ANOVA	0.026	Yes
	SNV	[0.609, 0.863, 0.037, 0.638, 0.684]	0.849	H	0.010	Yes
	SNV 1D 2-3-3	[0.217, 0.975, 0.327, 0.006, 0.299]	0.552	H	0.030	Yes
	SNV 1D 2-7-7	[0.140, 0.397, 0.091, 0.504, 0.505]	0.869	ANOVA	0.055	No
3	BSS SNV 1D 2-7-7	[0.084, 0.963, 0.967, 0.977, 0.428]	0.665	ANOVA	0.001	Yes
	RAW	[0.692, 0.306, 0.483, 0.053, 0.262]	0.711	ANOVA	0.005	Yes
	SNV	[0.468, 0.587, 0.208, 0.232, 0.359]	0.537	ANOVA	0.001	Yes
	SNV 1D 2-3-3	[0.415, 0.224, 0.327, 0.062, 0.200]	0.2328	ANOVA	0.061	No
	SNV 1D 2-7-7	[0.101, 0.110, 0.086, 0.886, 0.790]	0.792	ANOVA	0.019	Yes
4	BSS SNV 1D 2-7-7	[0.031, 0.071, 0.279, 0.311, 0.543]	0.802	H	0.011	Yes
	RAW	[0.021, 0.494, 0.967, 0.599, 0.198]	0.684	H	0.002	Yes
	SNV	[0.125, 0.113, 0.430, 0.480, 0.573]	0.444	ANOVA	2E-04	Yes
	SNV 1D 2-3-3	[0.741, 0.361, 0.566, 0.062, 0.434]	0.712	ANOVA	4E-04	Yes
	SNV 1D 2-7-7	[0.432, 0.719, 0.086, 0.864, 0.482]	0.753	ANOVA	2E-04	Yes
5	BSS SNV 1D 2-7-7	[0.690, 0.119, 0.101, 0.667, 0.052]	0.802	ANOVA	0.003	Yes
	RAW	[0.826, 0.758, 0.142, 0.412, 0.797]	0.546	ANOVA	0.008	Yes
	SNV	[0.517, 0.881, 0.309, 0.585, 0.359]	0.674	ANOVA	0.006	Yes
	SNV 1D 2-3-3	[0.641, 0.655, 0.785, 0.344, 0.034]	0.710	H	0.014	Yes
	SNV 1D 2-7-7	[0.497, 0.423, 0.464, 0.267, 0.025]	0.856	H	0.055	No
6	BSS SNV 1D 2-7-7	[0.420, 0.221, 0.482, 0.098, 0.937]	0.877	ANOVA	0.007	Yes
	RAW	[0.469, 0.573, 0.227, 0.126, 0.624]	0.876	ANOVA	1E-04	Yes
	SNV	[0.560, 0.216, 0.615, 0.675, 0.362]	0.868	ANOVA	3E-04	Yes
	SNV 1D 2-3-3	[0.139, 0.036, 0.665, 0.716, 0.606]	0.900	H	0.023	Yes
	SNV 1D 2-7-7	[0.326, 0.004, 0.626, 0.412, 0.719]	0.414	H	0.004	Yes

In Table S2, a robustness study of the most effective ML algorithms for each of the spectrum transformation algorithms employed has been conducted, categorized according to resampling methodologies. It should be noted that the study was conducted for each of the leading algorithms. However, according to the data presented in Table S1, it was not possible to determine the most effective algorithm, as significant differences could not be identified between all the optimized MLs.

Tables S3 and S4 depicts the statistical tests applied to spectral reflectance data. Table S3 identifies the most appropriate test for the optimized ML algorithms, and details whether there is significant differences

Table S2. Robustness study for each of the best ML models based on F1-Score with Absorbance. Abreviation: Res.=Resampling technique, Std=Standard deviation, CV=Variability coefficient, CI=Confidence interval, IQR=InterQuartile Range.

Res.	Transform	Best ML	Mean F1	Std	CV	CI95 Low	CI95 High	Median	IQR	Min	Max
1	BSS SNV 1D 2-7-7	SVC	0.92	0.08	0.09	0.82	1.01	0.94	0.09	0.79	0.98
	RAW	RF	0.89	0.06	0.06	0.82	0.96	0.92	0.01	0.79	0.93
	SNV	RF	0.88	0.05	0.06	0.82	0.94	0.89	0.01	0.81	0.94
	SNV 1D 2-3-3	SVC	0.91	0.07	0.07	0.82	0.99	0.94	0.04	0.79	0.96
	SNV 1D 2-7-7	SVC	0.90	0.06	0.07	0.82	0.98	0.92	0.04	0.79	0.95
2	BSS SNV 1D 2-7-7	SVC	0.92	0.06	0.06	0.85	0.99	0.92	0.05	0.85	1.00
	RAW	SVC	0.90	0.05	0.06	0.83	0.96	0.93	0.09	0.83	0.94
	SNV	RF	0.91	0.05	0.06	0.84	0.98	0.95	0.09	0.85	0.96
	SNV 1D 2-3-3	SVC	0.93	0.05	0.06	0.86	0.99	0.94	0.01	0.83	0.96
	SNV 1D 2-7-7	SVC	0.91	0.06	0.06	0.84	0.98	0.93	0.06	0.82	0.96
3	BSS SNV 1D 2-7-7	SVC	0.92	0.06	0.06	0.85	0.99	0.91	0.05	0.85	1.00
	RAW	RF	0.90	0.06	0.06	0.83	0.97	0.92	0.08	0.83	0.97
	SNV	RF	0.90	0.05	0.06	0.84	0.97	0.93	0.08	0.83	0.95
	SNV 1D 2-3-3	SVC	0.93	0.04	0.05	0.87	0.98	0.94	0.02	0.85	0.96
	SNV 1D 2-7-7	SVC	0.93	0.06	0.06	0.86	1.00	0.93	0.03	0.84	1.00
4	BSS SNV 1D 2-7-7	SVC	0.91	0.06	0.07	0.84	0.98	0.92	0.10	0.85	0.98
	RAW	RF	0.90	0.05	0.06	0.83	0.97	0.91	0.06	0.82	0.96
	SNV	RF	0.93	0.08	0.09	0.82	1.03	0.94	0.12	0.81	1.00
	SNV 1D 2-3-3	SVC	0.93	0.04	0.05	0.87	0.98	0.94	0.02	0.85	0.96
	SNV 1D 2-7-7	SVC	0.92	0.04	0.04	0.87	0.96	0.93	0.03	0.86	0.96
5	BSS SNV 1D 2-7-7	RF	0.92	0.06	0.07	0.84	1.00	0.96	0.08	0.83	0.97
	RAW	RF	0.90	0.07	0.07	0.82	0.99	0.94	0.10	0.82	0.97
	SNV	RF	0.90	0.08	0.09	0.80	1.00	0.93	0.10	0.78	0.97
	SNV 1D 2-3-3	SVC	0.93	0.05	0.05	0.87	0.99	0.95	0.03	0.85	0.98
	SNV 1D 2-7-7	SVC	0.92	0.06	0.06	0.85	0.99	0.94	0.08	0.83	0.97
6	BSS SNV 1D 2-7-7	SVC	0.90	0.06	0.07	0.82	0.98	0.94	0.10	0.82	0.96
	RAW	RF	0.90	0.08	0.09	0.80	1.00	0.94	0.09	0.78	0.96
	SNV	RF	0.92	0.08	0.08	0.82	1.01	0.95	0.08	0.81	1.00
	SNV 1D 2-3-3	SVC	0.90	0.06	0.07	0.83	0.98	0.92	0.07	0.82	0.97
	SNV 1D 2-7-7	SVC	0.91	0.04	0.05	0.86	0.96	0.91	0.07	0.86	0.95

(SD=*Yes*) between the experiments carried out. Table S4 presents a robustness study of the most effective ML algorithms for the different resampling and preprocessing experiments conducted.

As seen with VIS–NIR absorbance data and according to the statistical analysis carried out, it is difficult to determine the most effective algorithm because the differences between the optimized algorithms are minimal.

Table S3. Statistical study based on F1-Score with Reflectance. Abreviations: Res. = Resampling technique, H = Kruskal-Wallis test, and SD = Significant Differences.

Res.	Transform	Shapiro p-values	Levene p-value	Applied test	p-value	SD
1	BSS SNV 1D 2-7-7	[0.430, 0.910, 0.710, 0.430, 0.535]	0.167	ANOVA	0.000	Yes
	RAW	[0.383, 0.465, 0.961, 0.983, 0.966]	0.439	ANOVA	0.036	Yes
	SNV	[0.409, 0.228, 0.572, 0.876, 0.058]	0.264	ANOVA	0.018	Yes
	SNV 1D 2-3-3	[0.014, 0.023, 0.365, 0.153, 0.535]	0.444	H	8E-04	Yes
	SNV 1D 2-7-7	[0.959, 0.910, 0.710, 0.430, 0.535]	0.109	ANOVA	0.000	Yes
2	BSS SNV 1D 2-7-7	[0.228, 0.785, 0.269, 0.941, 0.682]	0.789	ANOVA	0.057	No
	RAW	[0.742, 0.162, 0.906, 0.776, 0.130]	0.914	ANOVA	0.032	Yes
	SNV	[0.946, 0.926, 0.788, 0.094, 0.447]	0.609	ANOVA	0.040	Yes
	SNV 1D 2-3-3	[0.142, 0.050, 0.972, 0.945, 0.778]	0.957	H	0.209	No
	SNV 1D 2-7-7	[0.662, 0.785, 0.269, 0.941, 0.149]	0.618	ANOVA	0.057	No
3	BSS SNV 1D 2-7-7	[0.386, 0.811, 0.223, 0.782, 0.399]	0.743	ANOVA	0.056	No
	RAW	[0.850, 0.910, 0.877, 0.611, 0.351]	0.603	ANOVA	0.045	Yes
	SNV	[0.133, 0.336, 0.969, 0.067, 0.389]	0.941	ANOVA	0.233	No
	SNV 1D 2-3-3	[0.179, 0.882, 0.864, 0.945, 0.905]	0.959	ANOVA	0.009	Yes
	SNV 1D 2-7-7	[0.584, 0.811, 0.223, 0.782, 0.360]	0.804	ANOVA	0.138	No
4	BSS SNV 1D 2-7-7	[0.005, 0.284, 0.008, 0.670, 0.601]	0.994	H	0.018	Yes
	RAW	[0.312, 0.987, 0.673, 0.776, 0.138]	0.132	ANOVA	1E-04	Yes
	SNV	[0.346, 0.169, 0.916, 0.463, 0.167]	0.958	ANOVA	0.019	Yes
	SNV 1D 2-3-3	[0.822, 0.427, 0.039, 0.945, 0.180]	0.913	H	0.064	No
	SNV 1D 2-7-7	[0.135, 0.284, 0.008, 0.669, 0.618]	0.992	H	0.057	No
5	BSS SNV 1D 2-7-7	[0.355, 0.012, 0.060, 0.171, 0.341]	0.789	H	0.027	Yes
	RAW	[0.736, 0.381, 0.800, 0.908, 0.103]	0.527	ANOVA	0.042	Yes
	SNV	[0.503, 0.969, 0.898, 0.089, 0.525]	0.600	ANOVA	0.008	Yes
	SNV 1D 2-3-3	[0.377, 0.821, 0.489, 0.279, 0.015]	0.879	H	0.127	No
	SNV 1D 2-7-7	[0.254, 0.012, 0.045, 0.171, 0.253]	0.857	H	0.034	Yes
6	BSS SNV 1D 2-7-7	[0.084, 0.797, 0.090, 0.982, 0.997]	0.982	ANOVA	0.000	Yes
	RAW	[0.034, 0.840, 0.878, 0.522, 0.354]	0.641	H	0.003	Yes
	SNV	[0.989, 0.276, 0.229, 0.548, 0.787]	0.749	ANOVA	0.105	No
	SNV 1D 2-3-3	[0.360, 0.447, 0.033, 0.614, 0.835]	0.955	H	0.009	Yes
	SNV 1D 2-7-7	[0.353, 0.797, 0.062, 0.982, 0.524]	0.883	ANOVA	0.000	Yes

Table S4. Robustness study for each of the best ML models based on F1-Score for Reflectance. Abreviation: Res.=Resampling technique, Std=Standard deviation, CV=Variability coefficient, CI=Confidence interval, IQR=InterQuartile Range.

Res.	Transform	Best ML	Mean F1	Std	CV	CI95 Low	CI95 High	Median	IQR	Min	Max
1	BSS SNV 1D 2-7-7	SVC	0.92	0.03	0.04	0.88	0.96	0.94	0.04	0.87	0.95
	RAW	SVC	0.89	0.04	0.04	0.84	0.93	0.89	0.04	0.84	0.94
	SNV	SVC	0.900	0.05	0.05	0.84	0.96	0.89	0.06	0.84	0.96
	SNV 1D 2-3-3	SVC	0.91	0.04	0.04	0.87	0.96	0.94	0.06	0.87	0.95
	SNV 1D 2-7-7	SVC	0.92	0.03	0.04	0.88	0.96	0.94	0.04	0.87	0.95
2	BSS SNV 1D 2-7-7	LGR	0.91	0.03	0.03	0.87	0.95	0.92	0.03	0.86	0.94
	RAW	RF	0.88	0.07	0.08	0.80	0.96	0.88	0.04	0.78	0.97
	SNV	RF	0.87	0.05	0.06	0.81	0.93	0.88	0.04	0.79	0.92
	SNV 1D 2-3-3	SVC	0.89	0.04	0.05	0.84	0.94	0.90	0.04	0.83	0.94
	SNV 1D 2-7-7	LGR	0.92	0.04	0.04	0.87	0.97	0.92	0.05	0.86	0.96
3	BSS SNV 1D 2-7-7	LGR	0.92	0.04	0.04	0.87	0.97	0.93	0.02	0.86	0.96
	RAW	SVC	0.87	0.04	0.05	0.82	0.92	0.87	0.04	0.81	0.91
	SNV	SVC	0.87	0.04	0.05	0.83	0.92	0.88	0.03	0.81	0.90
	SNV 1D 2-3-3	LGR	0.93	0.04	0.04	0.88	0.97	0.94	0.03	0.86	0.96
	SNV 1D 2-7-7	LGR	0.91	0.03	0.04	0.87	0.95	0.91	0.04	0.86	0.94
4	BSS SNV 1D 2-7-7	LGR	0.89	0.05	0.05	0.83	0.95	0.89	0.07	0.84	0.96
	RAW	SVC	0.87	0.04	0.04	0.83	0.92	0.87	0.04	0.82	0.91
	SNV	RF	0.87	0.04	0.05	0.81	0.92	0.87	0.03	0.81	0.92
	SNV 1D 2-3-3	LGR	0.89	0.03	0.03	0.86	0.93	0.89	0.04	0.86	0.93
	SNV 1D 2-7-7	SVC	0.89	0.05	0.05	0.83	0.95	0.89	0.07	0.84	0.96
5	BSS SNV 1D 2-7-7	LGR	0.92	0.04	0.04	0.87	0.97	0.92	0.01	0.88	0.98
	RAW	RF	0.87	0.06	0.07	0.79	0.95	0.88	0.06	0.79	0.96
	SNV	RF	0.88	0.05	0.06	0.82	0.95	0.88	0.04	0.81	0.94
	SNV 1D 2-3-3	LGR	0.91	0.04	0.05	0.86	0.96	0.91	0.07	0.87	0.96
	SNV 1D 2-7-7	SVC	0.91	0.05	0.05	0.85	0.97	0.94	0.08	0.85	0.96
6	BSS SNV 1D 2-7-7	SVC	0.92	0.04	0.04	0.87	0.97	0.92	0.03	0.87	0.97
	RAW	SVC	0.86	0.02	0.02	0.84	0.88	0.85	0.03	0.84	0.88
	SNV	SVC	0.87	0.04	0.05	0.82	0.91	0.88	0.04	0.80	0.90
	SNV 1D 2-3-3	SVC	0.91	0.04	0.05	0.86	0.97	0.92	0.06	0.85	0.96
	SNV 1D 2-7-7	SVC	0.92	0.04	0.04	0.87	0.97	0.92	0.03	0.87	0.97
